# Myoepithelioma of minor salivary gland - An immunohistochemical analysis of four cases

**DOI:** 10.1016/S1808-8694(15)31000-4

**Published:** 2015-10-19

**Authors:** Éricka Janine Dantas da Silveira, Antonio Luiz Amaral Pereira, Maria Carmen Fontora, Lélia Batista de Souza, Roseana de Almeida Freitas

**Affiliations:** a MS in Oral Pathology -UFRN, PhD student in Oral Pathology - UFRN; b PhD student at the Postgraduate Program in Oral Pathology - Federal University of Rio Grande do Norte; c PhD student at the Postgraduate Program in Oral Pathology - Federal University of Rio Grande do Norte; d PhD, Professor - Postgraduate Program in Oral Pathology - Federal University of Rio Grande do Norte; e PhD, Professor - Postgraduate Program in Oral Pathology - Federal University of Rio Grande do Norte

**Keywords:** minor salivary gland, immunohistochemical, myoepithelioma

## Abstract

**Introduction and Methods:**

We performed an immunohistochemical study in four cases of myopitheliomas with objective to realize a profile in respect of differentiation grade by the monoclonal antibodies CK14, vimentin and alph-SMA, besides to investigate the cell proliferation by anti-PCNA, besides, we compare the immunoreactive with glandular normal tissue.

**Results:**

In the glandular normal tissue the myoepithelials cells had shown expression for alpha-SMA and CK 14, while that in the ductals cells, only the presence of CK 14 was verified. All the cases was verified positivy for CK 14 and vimentin, however, CK 14 had been present only in epithelioid and fusiform cells, while that the vimentin revealed positive also in the cytoplasm of the plasmocytoid cells. alpha-SMA was not detected in the neoplasic cells. Immunopositivity for the PCNA was observed in more than 75% of the cellular component of the analyzed tumors, independent of the cellular type.

**Conclusions:**

We concluded that it did not have difference in the proliferative activity among the cellular types presents in the myoepitheliomas and, still, the results of this study suggest that the constituent cells of this neoplasia one really represent cells of the mioepitelial ancestry, but in different stages of differentiation.

## INTRODUCTION

Myoepitheliomas are rare benign neoplasias of the salivary gland, more commonly found in the parotid^1^, ^2^, being responsible for less than 7% of salivary gland tumors. It was first described in 1943^3^, ^4^. Such lesion shows a varied pattern of morphological growth, it may be solid, myxoid or reticular. It differs from the pleomorphic adenoma because it does not bear any ductal component^5^.

This tumor has varied cell morphology, being fusiform, plasmocytoid, epidermoid or clear cells^6^. Some studies have shown that fusiform cells have some muscular differentiation because they react to a-SMA and vimentin^4^, while other investigations did not show any muscular differentiation in the plasmocytoid cells^4^, ^7^. According to Jaeger et al. (1997), these cells could have origins other than myoepithelial, and they lost or had changed their capacity to express muscular evidence markers.

Besides the many phenotypes myoepithelioma cells may have, some authors report that fusiform and clear cells have a higher prolipherative capacity, when compared to the plasmocytoid cells, and they also stated that the production of myxoid material would be related to the low prolipherative activity of these tumors^9^.

Medical literature mentions the use of different markers for histogenetic-related prolipherative activity both in the normal salivary gland and also in the glandular benign and malignant tumors. Thus, we should use immunohistochemistry to analyze differentiation patterns and the prolipherative activity of the different cell types present in salivary gland myoepitheliomas.

## MATERIALS AND METHODS

We selected 4 cases of small salivary gland myoepitheliomas from the files of the Pathology lab of the Oral Pathology Department of the Federal University of Rio Grande do Norte. Paraffin-bounded specimens were cut in 5mm thickness slices and hematoxylin-eosin was used for the cellular morphology analysis.

**Chart 1:**
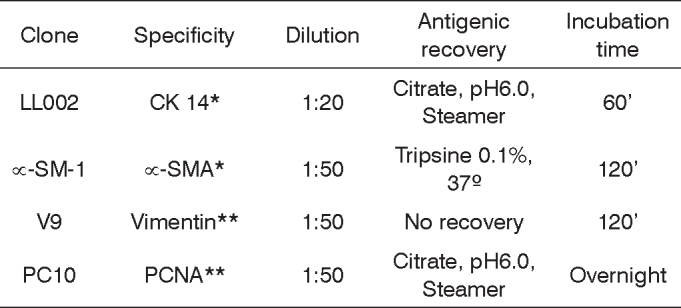
Antibodies used.

An immunohistochemical study by the streptavidine-biotin technique was carried out using antibodies against vimentin, a-SMA, CK 14 and PCNA (Cell Proliferation Nuclear Antigen). Chart 1 lists the clones, antigenic recovery, dilution, incubation time and manufacturers of the antibodies used.

All the material selected was fixed in formaldehyde and embedded in paraffin, histological cross-sections of 3μm were made and placed on slides adhered by 3-aminopropyltriethoxy-silane (Sigma Chemical CO., St. Louis, MO, USA). The histology cross-sections were deparaffined in xylol, rehydrated in an alcohol sequence up to water and washed in two distilled water vials for 5 minutes each. Endogenous peroxidase was blocked by hydrogen peroxide 20vol, flushed with water and incubated in TRIS-HCL (Trishydroximethil-aminomethane), pH 7.4 for 10 minutes. The cross-sections were incubated with anti-mouse monoclonal antibody, diluted in a TRIS-HCL buffer solution (Chart 1), for incubation with the streptoavidine-Biotin complex, in a 1:100 dilution for 30 minutes. For development purposes, we used a 0.03% diaminebenzidine cromogen solution, diluted in TRIS-HCL added to 0.6ml of 20vol hydrogen peroxide in a dark chamber for 3 minutes. For counter-coloring we used Mayer Hematoxylin for 10 min, flushing in water after each step. To finish the process we used alcohol for dehydration and diaphanization in xylol for slide preparation with Permount.

Fragments of normal salivary gland were used as internal positive control and for comparative purposes.

The immunopositiviness analysis was carried out by two examiners at different times, in a double-blinded study through light microscopy, and all the Brown colored cells in their cytoplasm or nucleus were considered positive (PCNA). Thus we investigated the presence or absence of markers, assigning the following scores: - (no marker); + (focal marker, less than 10% of cells marked) and ++ (diffuse marking).

## RESULTS

Table 1 lists the patients’ clinical data.

**Table 1:** Clinical data of the myoepithelioma cases studied.

Case	Gender	Age	Anatomic location	Clinical diagnosis	Size
1	Male	39 years	Hard palate	Adenoma pleomorphic	2,5 cm
2	Male	80 years	Upper lip	Lipoma	0,5 cm
3	Female	30 years	Hard palate	Adenoma pleomorphic	0,5 cm
4	Male	31 years	Hard palate	Adenoma pleomorphic	2,0 cm

### Morphological Results

Tumors were well circumscribed, and we frequently found a fibrous connective tissue capsule surrounding the specimens. The four tumors presented a predominantly solid growth and organizational patterns. The specimens were made up of nests of cohesive and non-cohesive cells in a matrix that varied between hyaline and myxoid. Tumor cells showed different morphologies, frequently fusiform, polygonal of eosinophilic cytoplasm (epithelioid) and, sometimes, with hyper chromatic nucleus. No tumor had necrotic areas; although in one case we did find squamous metaplasia and calcifications.

### Immunohistochemical results

**Figure 1:**
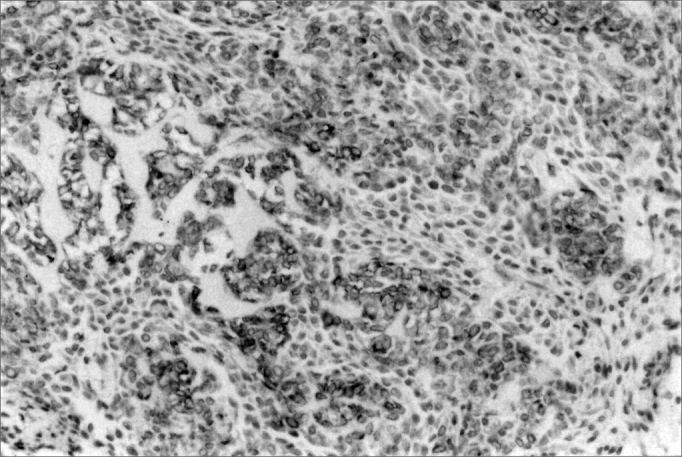
CK 14 expression in epithelioid and fusiform cells in small salivary gland myoepitheliomas (SABC, 200x).

**Figure 2:**
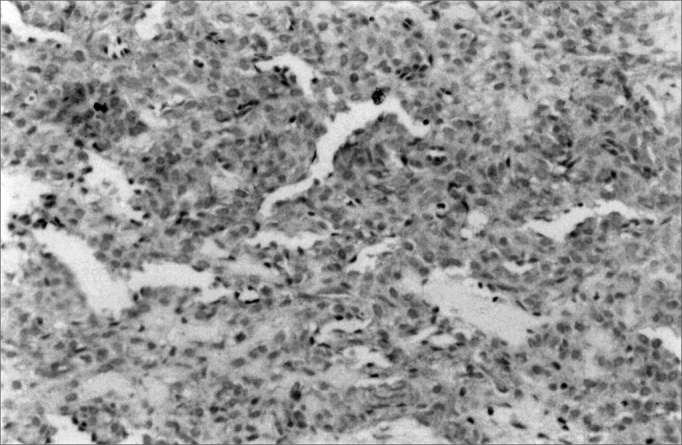
Vimentin immonomarking in small salivary gland myoepithelioma (SABC, 200x).

The immunohistochemical marking for the analyzed antibodies may be seen in Figures 1 and 2. All the cases had immunopositiviness for CK 14 and vimentin. However, CK 14 was present only in epithelioid and fusiform cells, while vimentin was also present in the cytoplasm of plasmocytoid cells. a-SMA was not detected in neoplastic cells, although it was present in the blood vessels, and they were used as internal positive control.

Immunopositiviness for PCNA was seen in more than 75% of cell components belonging to the analyzed tumors, regardless of cell type and the amount of stroma present.

Next to 2 of the 4 myoepitheliomas analyzed there was normal glandular tissue, predominantly made of ducts and mucous acini. In this tissue, the myoepithelial cells showed markers for a-SMA and CK 14. Immune marking for CK 14 was detected in ductal cells. Table 2 lists the immunoreactions for the analyzed myoepithelioma antibodies.

**Table 2:** Immunohistochemical findings in our cases of small salivary gland myoepitheliomas.

Case	CK 14	∝-SMA	Vimentina	PCNA
1	+	-	++	++
2	+	-	++	++
3	+	-	+	++
4	+	-	+	++

-: no mark, +: focal mark, ++ diffuse and intense mark

## DISCUSSION

The benign myoepithelioma of salivary gland is a rare neoplasia of myoepithelial nature, made up of fusiform, epithelioid, plasmocytoid and clear cells^10^. For some authors such as Simpson et al. (1995) this neoplasia is but a rare type of pleomorphic adenoma. However, according to Dardick et al. (1995), it is different from the pleomorphic adenoma because it bears little or no ductal component.

An interesting aspect about myoepitheliomas is the scarce information we have about its biological behavior, because of its low incidence rates. Some authors consider it to be more aggressive than pleomorphic adenomas^11^, while another investigation noticed, through PCNA expression, that there were no differences as far as prolipherative activity is concerned between myoepitheliomas and pleomorphic adenomas^9^.

Ogawa et al. (1993) reported that cell kinetics is related to the biological behavior of many tumor types, and they noticed in their study that myoepitheliomas made up of fusiform, epithelioid and clear cells have greater rates of PCNA cells when compared to the plasmocytoid type, thus confirming the low prolipherative activity in this type. Such findings disagree from the ones in the present investigation because here the PCNA is marked in over 75% of the tumors analyzed, regardless of cell type.

Myoepithelial cells, components of this neoplasia, are also part of many other salivary gland tumors. According to reports from Araújo et al. (1994) and Batsakis and El-Naggar (1999) these cells are involved in many processes related to neoplastic growths, such as differentiation of tumor cells, synthesis of basal membrane and maspin tumoral suppressor, besides also inhibiting invasion and angiogenesis. Capuano and Jaeger (2004) reported the presence of matrix constituents, such as laminin, for example, that can induce morphologic changes in myoepitheliomas, causing a phenotype made up of plasmocytoid cells.

According to Jaeger et al. (1997) a reasonable proportion of fusiform cells in the myoepitheliomas point toward muscular differentiation, reacting positively for a-SMA and vimentin, and these are called “myoepithelial like”.

Reports by Ellis and Auclair (1996) state that except for fusiform cells and some epithelioids, the true nature of myoepitheliomas plasmocytoid is still unclear. Jaeger et al. (1997) also reported that these cells did not show any evidence of muscular differentiation. Ogawa et al. (2003) believe that these cells are originated from luminal cells and not myoepithelial, also adding that plasmocytoid-cell tumors could be classified as adenomas or plasmocytoid adenocarcinomas.

Immunohistochemical markers for myoepithelial cells have included protein S-100, GFAP, cytokeratin and vimentin, and also a-SMA, which is associated to myoepithelial cells of the normal salivary gland^7^. Araújo et al. (1994) consider vimentin a marker of neoplastic myoepithelial cell. However, studies performed by this group in 2001^14^, suggest that this protein does not happen solely in neoplastic myoepithelial cells, since it may be present also in other cell types originated from the intercalate duct.

According to Hornick and Futcher (2004), neoplastic myoepithelial cells frequently lose the expression of muscular differentiation markers, when the immunoreactivity to these markers is not required in order to confirm myoepithelial differentiation.

Thus, the low expression of muscle tissue markers has led the authors to consider plasmocytoid myoepitheliomas as true myoepitheliomas, despite the fact that Franquemont and Mills (1993) do not believe in the myoepithelial nature of this cell type, suggesting that the plasmocytoid subtype would not be a type of myoepithelioma.

Having so much debate in the literature as to the true nature of myoepithelioma cells, the present study analyzed the differentiation and the nature of small salivary gland myoepithelioma cell components, using epithelial cell differentiation markers such as CK 14 and of muscle tissue differentiation such as a-SMA and vimentin.

Cytokeratin are proteins of the intermediary filaments of epithelial cells associated to the differentiation and organization of the cytoskeleton, and they are expressed in specific epithelial cells depending on their differentiation stage. According to Ogawa et al. (1999) and Ogawa et al. (2000), salivary gland myoepithelial cells Express CK 5 and 14; stressing the fact that these do not serve as specific markers for such cells because they are also expressed by basal ductal cells, where CK 18 and 19 are also present.

CK 14 was present in myoepithelial cells (non-luminal), of fusiform, epithelioid and plasmocytoid morphology in the pleomorphic adenomas studied by Ogawa et al. (2003), and it disagrees from the findings of the present investigation in which CK 14 was only immunomarked in fusiform and epithelioid cells, and such fact also disagrees from the findings by Araújo et al. (2001) where CK 14 and 19 marks were found in plasmocytoid cells and not in fusiform cells.

The vimentin found in all the cell types of the present study was also present in all myoepithelioma cell types analyzed in the investigation by Araújo et al. (2001) and, occasionally, in non-luminal cells of the pleomorphic adenomas studied by Ogawa et al. (2003). The latter authors stress the hypothesis that this protein is not an exclusive maker of the neoplastic myoepithelial cell, justifying that it is necessary for the migration of epithelial cells in both physiologic and pathologic processes, and vimentin is also present during the development after this process in some segments of the normal salivary gland.

The lack of a-SMA in the cases hereby studied and the immunoreactivity for vimentin were also detected by Savera et al. (1997) and Ogawa et al. (2003), respectively.

With such findings, we believe that all morphologic types of myoepithelioma cells represent cells of the myoepithelial strain in different stages of evolution, disagreeing from the conclusions reached by Ogawa et al. (2003). The controversies present in the literature regarding the expression of muscle-tissue markers in fusiform, epithelioid and plasmocytoid cells may be justified by the fact that such cells may show different stages of differentiation, or they may have lost or changed their capacity of producing muscle-tissue markers. Moreover, it may be suggested that the low muscle-tissue differentiation seen “in vivo” in some cells which make up the myoepitheliomas may be caused by the inhibitory process mediated by the extracellular matrix.
